# Detection of active proteasome structures in brain extracts: proteasome features of August rat brain with violations in monoamine metabolism

**DOI:** 10.18632/oncotarget.20208

**Published:** 2017-08-10

**Authors:** Pavel A. Erokhov, Yulia V. Lyupina, Alexandra S. Radchenko, Anna A. Kolacheva, Yulia O. Nikishina, Natalia P. Sharova

**Affiliations:** ^1^ Laboratory of Biochemistry of Ontogenesis Processes, N.K. Koltsov Institute of Developmental Biology of Russian Academy of Sciences, Moscow, Russia; ^2^ Laboratory of Neural and Neuroendocrine Regulations, N.K. Koltsov Institute of Developmental Biology of Russian Academy of Sciences, Moscow, Russia

**Keywords:** proteasome structure, immune proteasomes, rat brain, violations in monoamine metabolism, native electrophoresis

## Abstract

The aim of this work was to detect changes in proteasome pools of brain parts of August rats with monoamine metabolism violations in comparison with that of control Wistar rats. To reveal active proteasome structures, a method of native electrophoresis for the analysis of crude tissue fractions was developed. By means of this method and following Western blotting, the most pronounced changes in reorganization of proteasome structures were detected in proteasome pool of the brain cortex of August rats. Main findings are the enhanced expression of immune proteasome subtypes containing proteolytic subunit LMP2 and activator PA28αβ as well as immune proteasome subtypes containing proteolytic subunit LMP7 and activator PA700 and simultaneously decreased expression of subtypes with subunit LMP2 and activator PA700 in the brain cortex of August rats compared to that of Wistar rats. These results were indirectly confirmed by SDS PAGE method followed by Western blotting, which showed the increased quantities of immune subunits and proteasome activators in the brain cortex of August rats compared to that of Wistar rats. Immune proteasomes were revealed by immunohistochemistry in neurons, but not in glial cells of August and Wistar rat cortex. The detected reorganization of proteasome pools is likely to be important for production of special peptides to provide the steady interaction between neurons and adaptation of central nervous system to conditions caused by monoamine metabolism deviations.

## INTRODUCTION

The study of adaptive mechanisms developing in the brain under stress conditions is tightly related to the elucidation of neural plasticity phenomenon. Of great interest is the stress provoked by changes in the content of monoamines especially serotonin. Serotonergic mechanisms play an important role in the regulation of anxiety behavior in mammals [1–3]. At the early stages of the development of central nervous system, synapse formation and elimination depend on serotonin [4]. The inbred line of August rats is characterized by genetically determined disorders in monoamine metabolism and therefore it represents the convenient model for the study of effects of these disorders. In the motor cortex of August rats, the level of biogenic amines and their metabolites is known to be higher than in the midbrain reticular formation in contrast to Wistar rats [5, 6]. Despite of this imbalance, the exchange between the cortex and subcortical structures that manages function of the cerebral cortex, in August rats remains intact [7]. August rats show higher inherent basal activity of stress-limiting systems (NO synthase and heat shock proteins, HSPs) accompanied by the increased resistance to the acute emotional stress in comparison with Wistar rats [8–10].

Other key molecular participators important for adaptation of the brain to deviations in monoamine metabolism remain poorly studied and understood.

It is known that proteasomes, multicatalytic proteases, hydrolyze up to 90% of proteins in cells and regulate numerous cellular processes. Proteasomes participate in adaptive mechanisms under oxidative stress and stress influenced by the absence of major histocompatibility (MHC) class I molecules [11–14]. In this regard, we focused our attention on proteasome study in the brain of August rats.

Cellular proteasome pool consists of multiple forms differed in the combination of proteolytic subunits and regulators. 20S proteasome, or 20S core particle, has a barrel-shaped structure that is composed of four rings of seven subunits each, two outer rings of ɑ subunits and two internal rings of β subunits [15]. Hydrophobic N-ends of ɑ subunits form barrier preventing the casual penetration of proteins into the proteolytic chamber. Proteolytic activities are associated with the internal β rings. Catalytic subunits β1, β2, and β5 demonstrate caspase-like (CL) (also termed peptidyl-glutamyl peptide-hydrolyzing), trypsin-like, and chymotrypsin-like (ChTL) activities respectively. Mammals have special proteasome forms, immune proteasomes. They contain proteolytic subunits LMP7(β5i), LMP2(β1i), and MECL1(β2i) instead of subunits β5, β1, and β2 of constitutive proteasomes. Replacement of constitutive subunits by immune ones takes place during new proteasome assembling. Subunits LMP2 and MECL1 may be integrated together and independently from LMP7. Although incorporation of subunit LMP7 is facilitated by subunits LMP2 and MECL1, this process can also take place without them [16, 17]. Both immune and constitutive forms are capable of hydrolyzing the foreign (mutant or viral) and own cellular proteins [18]. The immune proteasomes, like constitutive ones, display ChTL, CL and trypsin-like activities. At the same time, the immune proteasomes have the altered cleavage site preference due to the change in the conformation of substrate binding pockets that affects the structure of the peptides produced [19]. On the whole, in the immune proteasomes ChTL and trypsin-like activities are higher and CL activity is lower than in the constitutive proteasomes. It allows the immune proteasomes to generate antigen epitopes for MHC class I molecules or other special peptides in a large quantity.

20S proteasome is the smallest functional structure of 750 kDa able to cleave a number of substrates including proteins with damaged conformation under oxidative stress and several viral proteins [11, 12, 20, 21]. The specificity of ubiquitinated protein hydrolysis is ensured by 26S proteasome of approximately 2.5 MDa consisting of 20S core particle and PA700 (19S) activator [22, 23]. After recognition and binding of ubiquitinated proteins, PA700 activator carries out their deubiquitination and ATP-dependent unfolding. Multifunctionality of this activator is provided by 20-21 subunits that organize its structure. On the whole, 26S proteasomes secure the protein rotation in cells and elimination of damaged proteins.

The specificity of proteasome functioning depends not only on PA700 activator and immune subunits, but also on activators PA28 (11S) and PA200, flanking proteasome 20S core particle. Cytoplasmic activator PA28ɑβ, containing 3ɑ and 4β (or 4ɑ and 3β) subunits of 28 kDa each, stimulates hydrolysis of unstructured proteins and polypeptides of middle and small length. Proteasomes with PA700 and PA28 activators may produce special peptides for MHC class I molecules even in the absence of proteolytic immune subunits [18, 24, 25]. Activator PA200, the protein of 200 kDa, regulates proteasome proteolytic activity by opening of ɑ canal for not long substrates. It is also possible that activator PA200 facilitates the release of hydrolysis products [26].

Different sets of multiple proteasome forms provide the specific functions of each tissue and organ. The aim of this work was to detect changes in proteasome pools of the brain parts of August rats with monoamine metabolism violations in comparison with that of control Wistar rats. This aim was achieved with the use of native electrophoresis method developed for the analysis of proteasomes in crude tissue fractions.

## RESULTS AND DISCUSSION

### Characteristic of monoamine metabolism and HSP70 expression in the brain parts of August rats

First of all, we confirmed the presence of changes in the content of serotonin and dopamine in the brain parts of August rats taken in the experiment, in comparison with that of Wistar rats. The content of both monoamines in the cortex and striatum in August rats was higher than in Wistar rats (Table 1). The lower ratio of the content of homovanillic acid (dopamine metabolite) and dopamine, as well as 5-hydroxyindoleacetic acid (serotonin metabolite) and serotonin in the cortex and striatum of August rats indicates the less rate of dopamine and serotonin metabolizing in these brain parts of August rats compared to Wistar rats (Table 1). In the brainstem of August rats, the level of dopamine, but not serotonin, was higher in relation to that of Wistar rats due to the less intensity of metabolizing processes detected by the ratio of the quantity of dopamine metabolites, 3,4-dihydroxyphenylacetic acid and homovanillic acid, to dopamine (Table 1).

**Table 1:** Content of serotonin, dopamine and their major metabolites in brain parts of Wistar and August rats

Monoamine	Content of monoamine, pmol/mg of tissue					
	In cortex		In striatum		In brainstem	
	W	A	W	A	W	A
DA	0.4 ± 0.1	0.6 ± 0.1*	4.5 ± 1.1	13.5 ± 1.3*	0.2 ± 0.1	0.4 ± 0.1*
DOPAC	0.2 ± 0.1	0.3 ± 0.1	4.9 ± 1.3	13.5 ± 1.2*	0.1 ± 0.1	0.1 ± 0.1
HVA	0.2 ± 0.1	0.1 ± 0.1	3.5 ± 0.4	3.8 ± 0.3	0.1 ± 0.1	0.1 ± 0.1
5-HT	1.0 ± 0.1	1.9 ± 0.2*	1.0 ± 0.1	1.6 ± 0.2*	1.4 ± 0.2	1.6 ± 0.1
5-HIAA	2.8 ± 0.2	3.5 ± 0.2*	3.7 ± 1.1	3.8 ± 0.1	2.2 ± 0.2	2.5 ± 0.2
	Ratio of monoamine content					
DOPAC/DA	0.5±0.1	0.5±0.1	1.1±0.3	1.0±0.3	0.5±0.1	0.3±0.1*
HVA/DA	0.5±0.1	0.2±0.1*	0.8±0.3	0.3±0.1*	0.50±0.1	0.3±0.1*
5-HIAA/5-HT	2.8±0.2	1.8±0.2*	3.7±1.1	2.4±0.2*	1.6±0.2	1.6±0.2

A, August rats; W, Wistar rats; DA, dopamine; DOPAC, 3,4-dihydroxyphenylacetic acid; HVA, homovanillic acid; 5HT (5-hydroxytryptamine), serotonin; 5-HIAA, 5-hydroxyindoleacetic acid.

Standard deviation from the average magnitude is shown. *Reliable difference from corresponding control magnitude (*p* < 0.05, n = 6).

**Figure 1:**
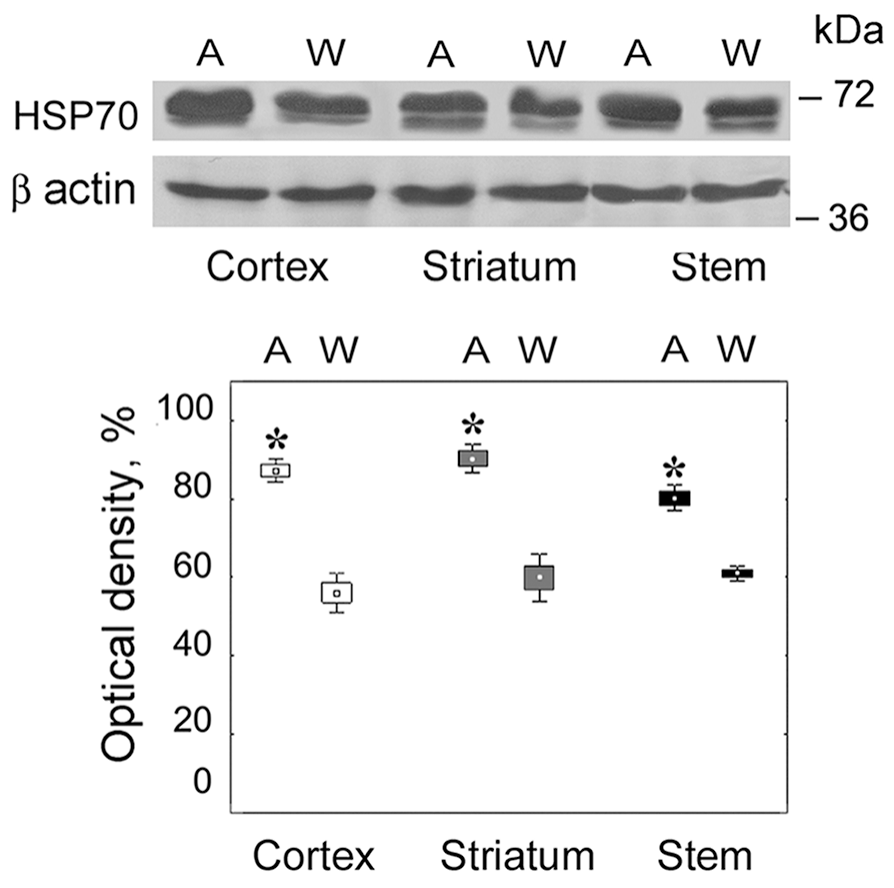
Content of HSP70 in brain parts of August and Wistar rats On the top, Western blots of HSP70 with the use of corresponding antibodies. Molecular mass of standard protein markers is shown. On the bottom, relative subunit quantities as percentage from the maximum magnitude normalized to β actin level and presented as means and SEM. *Reliable difference from control magnitude (*p* = 0.02, n = 8). A, August rats; W, Wistar rats.

We revealed the enhanced level of stress-limiting HSP70 protein in all studied brain areas of August rats in comparison with Wistar rats (H(1,32) = 5.23; *p* = 0.02) (Figure 1). This result indicated the stress conditions in the brain of August rats.

Thus, the brain of August rats taken in the experiment was characterized by the presence of the stress associated with monoamine metabolism deviations.

### Expression patterns of proteasome subunits in the brain parts of August rats

To detect changes in the expression of proteasome subunits, the standard approach with the use of SDS PAGE followed by Western blotting was applied. Only the protein content of proteasome subunits (not mRNA content) was determined, because mRNA levels were not well correlated with the amounts of proteasome proteins, suggesting a specific regulation of proteasome subunit synthesis [27]. The relative content of proteasome proteolytic constitutive subunits β1, β5 and immune subunits LMP2, LMP7 was evaluated with utilizing the antibodies to these subunits. Because of the joint incorporation of LMP2 and MECL1 subunits into proteasome structure [16], it was enough to analyze LMP2 expression patterns for the evaluation of changes in the expression of both subunits. The level of activator PA200 was detected with the use of antibodies to this protein. The expression patterns of activators PA700 and PA28ɑβ were studied with the application of antibodies to subunits Rpt6 and PA28ɑ included in the structures of these activators respectively. The total proteasome pool was studied by means of combined antibodies to subunits ɑ1,2,3,5,6,7 incorporated in all proteasome forms.

**Figure 2:**
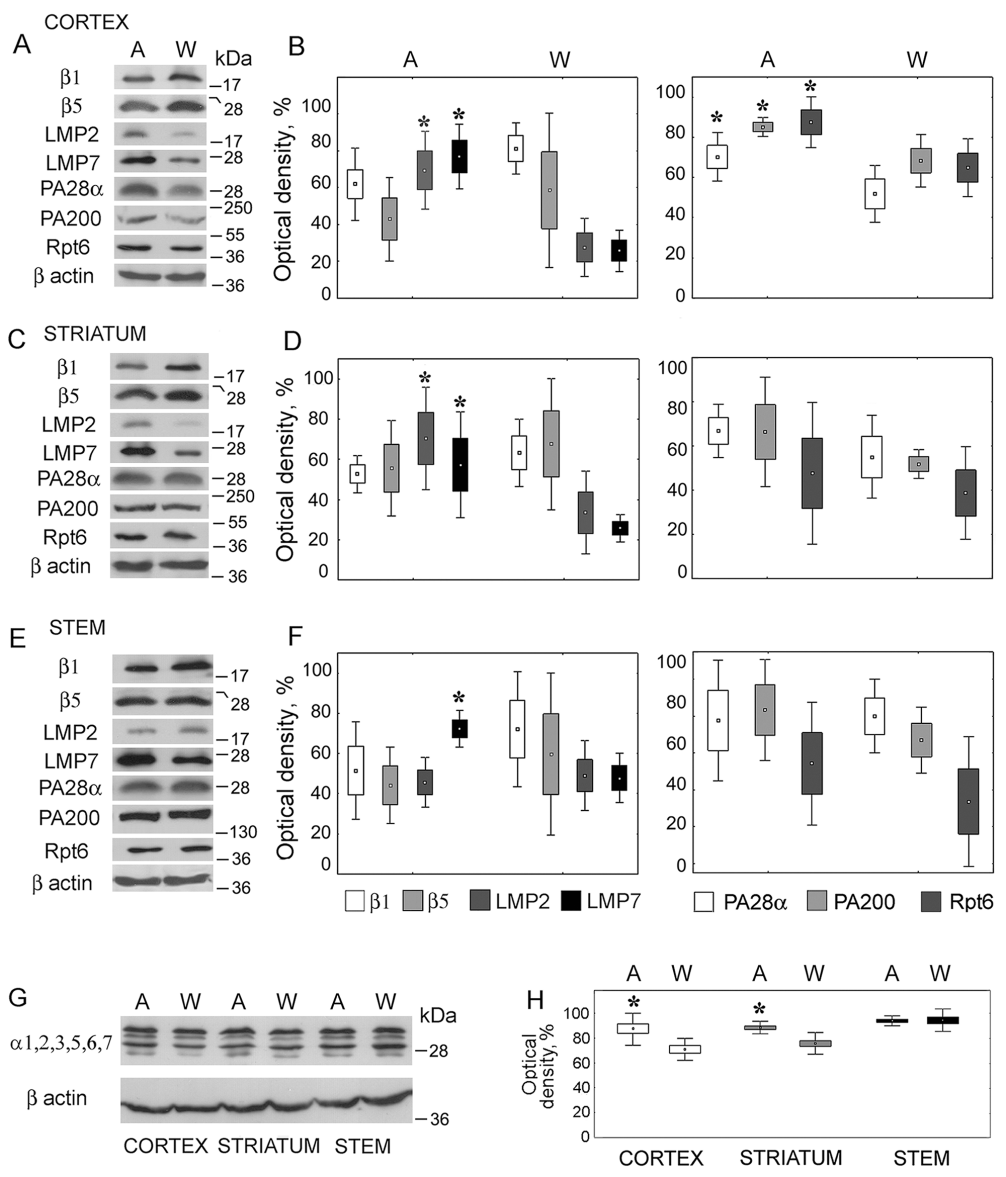
Expression of proteasome and activator subunits in brain parts of August and Wistar rats **(A, C, E, G)** Western blots of proteasome and activator subunits with the use of corresponding antibodies. Molecular mass of standard protein markers is shown. **(B, D, F, H)** The relative subunit quantities as percentage from the maximum magnitude normalized to β actin level and presented as means and SEM. *Reliable difference from control magnitude (*p* < 0.05, n = 8). A, August rats; W, Wistar rats.

Proteasome subunit levels were dependent on the brain structure and rat strain (H(1,18) = 0.235; *p* = 0.015). The most marked changes in the proteasome pool were revealed in the brain cortex of August rats compared to that of control Wistar rats. In this brain part, the expression of immune proteolytic subunits LMP7 (H(1,8) = 5.46; *p* = 0.01) and LMP2 (H(1,8) = 5.43; *p* = 0.02) was three times higher (Figure 2A and 2B). The expression of activators PA700 (H(1,8) = 3.88; *p* = 0.04), PA28ɑβ (H(1,8) = 5.36; *p* = 0.03) and PA200 (H(1,8) = 5.46; *p* = 0.01) was also enhanced but to the less degree, up to 40%.

In the striatum of August rats, the content of LMP7 (H(1,8) = 5.33; *p* = 0.02) and LMP2 (H(1,8) = 5.36; *p* = 0.03) subunits was two times higher (Figure 2C and 2D). The expression of proteasome activators showed the tendency to increase, but reliable differences were not detected.

In the stem, only the expression of LMP7 subunit (H(1,8) = 3.85; *p* = 0.04) was higher (by 50%) in comparison with control (Figure 2E and 2F).

The level of the total proteasome pool was increased by 20% and 25% in the striatum and cortex of August rats respectively, in the stem it was identical in August and Wistar rats (Figure 2G and 2H). At the same time, there was no expected reliable decrease, except of the tendency to decrease, in the content of constitutive subunits β1 and β5 replaced by immune subunits LMP2 and LMP7 (Figure 2A–2F). This apparent contradiction may be explained by the small portion of the immune subunits in comparison with the content of constitutive ones in the total proteasome pools. So, the essential increase of the quantity of the immune subunits is reflected in unessential and unreliable decrease of the level of the constitutive subunits.

**Figure 3:**
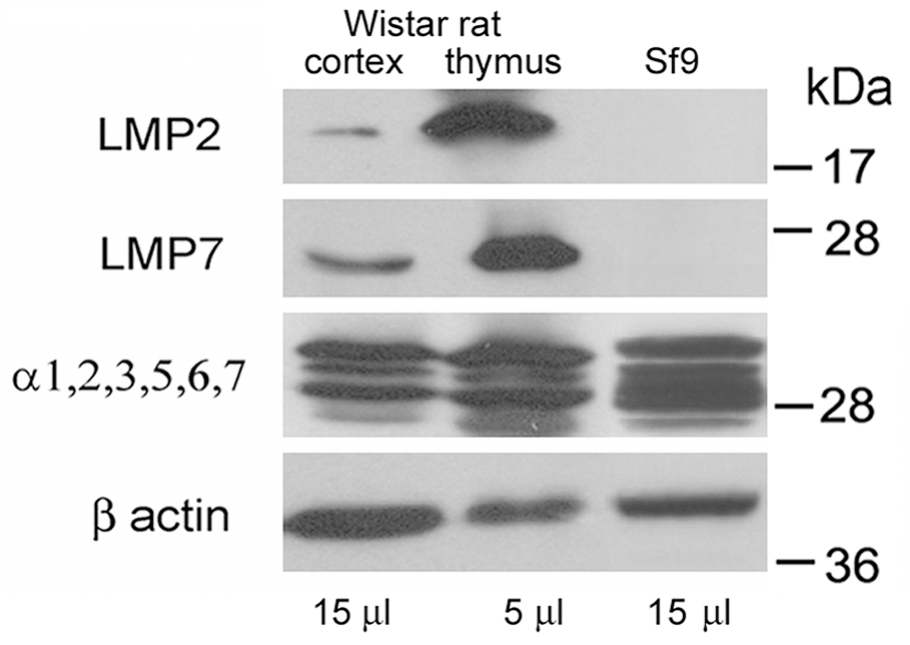
Western blots of proteasome subunits of rat organs and insect cells Extract of Wistar rat thymus was used as a positive control; extract of *Spodoptera frugiperda* Sf9 cells was used as a negative control. Molecular mass of standard protein markers is shown.

Note that the immune proteasomes are known to be present mainly in the organs of the immune system. So, we compared the quantities of LMP7 and LMP2 subunits in extracts of rat brain cortex and thymus. At the same time, thymus extract may serve as a positive control of the immune subunit presence. In the brain cortex, much lesser quantities of the immune subunits were detected (Figure 3). Normalized to β actin content, the quantities of LMP7 and LMP2 subunits were 8 and 15 times less in the brain cortex than in thymus. Normalized to the total proteasome pool, the content of these subunits was respectively 4 and 7 times less in the brain cortex than in thymus. In this regard, the appropriate choice of tissue extract volume, that would depend on the peculiarities of the proteasome pool, is important for the immune proteasome investigation. Besides, extract of insect Sf9 cells prepared as earlier described [28] served as a negative control of the immune subunit presence.

The data obtained showed the interesting information about the brain proteasome subunits of August and Wistar rats, but they did not allow us to realize the characteristics of the individual proteasome subtypes in the brain of these rats. We took into account the possibility that not all proteasome subunits detected in this experiment were incorporated in proteasome structure. So, preliminarily we had to develop native electrophoresis method appropriate for the analysis of proteasomes in crude tissue fractions. The investigation of impure tissue extracts allows us to discover the multiplicity of proteasome structures more correctly compared to purified samples whose preparation is often accompanied by the loss of some proteasome forms.

### Detection of special conditions for native electrophoresis

**Figure 4:**
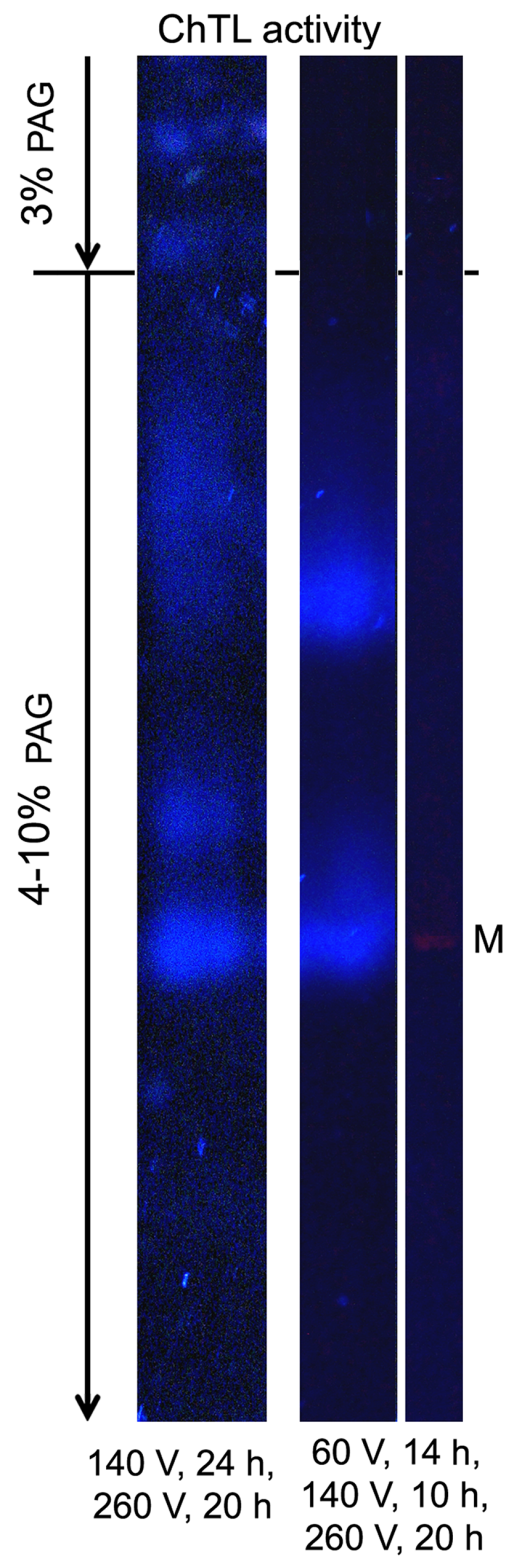
Proteasome ChTL activity of liver extract in the gel after native electrophoresis under different voltage conditions Thyroglobulin (670 kDa), labeled by the dye Cy-3.5, was applied as a marker (M) of molecular mass. The distribution of activity in the gel was identical in all experiments (n = 6).

The method was modified on the basis of published protocol for native electrophoresis [28]. Conditions for native electrophoresis were detected with the use of rat liver extract as a fraction most contaminated by microsomes. Nucleic acids and microsomes in probes impede the entry of high molecular mass proteins into gel and constitute the main problem of crude extract electrophoresis at standard initial voltage of 140 V. Under these conditions, proteasomes nonspecifically combined with microsomes and / or nucleic acids are distributed throughout the gel beginning from the start position (Figure 4). We solved this problem by low initial voltage of 60V applied for 14 h (Figure 4).

Note that proteasome activity in the gel looked as blurred zones formed by the diffusion of short reaction products.

### Expression patterns of proteasome subtypes in the brain cortex of August rats

**Figure 5:**
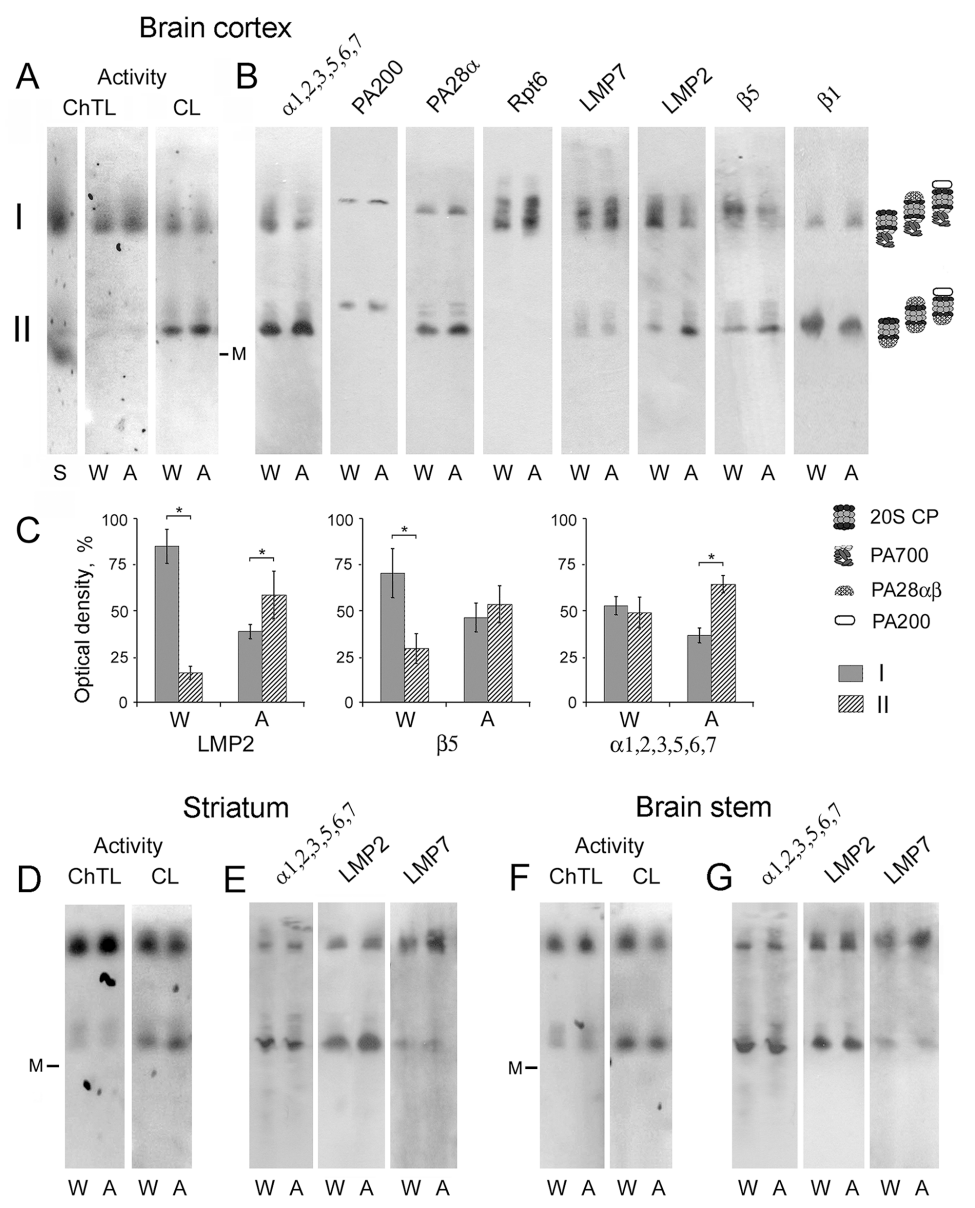
Electrophoresis of brain extracts of Wistar and August rats in native conditions **(A, D, F)** Proteasome ChTL and CL activities of the brain cortex, striatum and stem in the gel. Thyroglobulin (670 kDa), labeled by the dye Cy-3.5, was used as a marker (M) of molecular mass. Extract of Wistar rat liver was used as a standard (S). **(B, E, G)** Western blots of proteasome and activator subunits with the use of corresponding antibodies. **(C)** The relative subunit quantities in discrete zones I and II as percentage from total quantity in zone I + zone II after electrophoresis of the brain cortex proteins. Error bars, SEM. *Data reliably differ from control at *p* < 0.05, n = 6. A, August rats; W, Wistar rats.

By means of method of native PAGE followed by Western blotting, six active proteasome forms differed in the combination of activators were revealed in the brain parts of August and Wistar rats (Figure 5). We conditionally marked two zones, I and II, in the gel. In zone I, active proteasome structures of high molecular mass due to the presence of activator PA700 are detected. In fact, they represent different forms of 26S proteasomes: PA700-20S, PA700-20S-PA28ɑβ, PA700-20S-PA200. Zone II contains active proteasome structures with lower molecular mass: PA28ɑβ-20S, PA28ɑβ-20S-PA28ɑβ and PA28ɑβ-20S-PA200.

Earlier, under other electrophoresis conditions three proteasome forms were detected in the rat cortex: two forms coupled with PA700 activator, and a form free of PA700 activator [29]. It is very probable that short electrophoresis time of 4.5-6.5 h, applied in this work, was insufficient for the high resolution of proteasome structures. In other study, the use of electrophoresis time of 41 h for the division of partially purified proteasome pool of rabbit reticulocytes into separate proteasome forms allowed the authors to achieve high resolution [30].

Of great interest is the fact of the absence of free 20S proteasomes in the brain. This is discovered firstly by the location of 670 kDa marker (Figure 5A, 5D and 5F), secondly by the comparison of electrophoretic lanes with brain samples and standard sample of liver extract (Figure 5A) where 20S proteasomes have been detected earlier [31]. Besides, there is no proteasome band (labeled by mAb to ɑ1,2,3,5,6,7 subunits) in the gel free of any proteasome activator (Figure 5B). Taking into account the major role of 20S proteasomes as the remover of damaged proteins [11, 12], it is consistent to conclude that the brain possesses effective stress-limiting systems which allow it to function without this proteasome form.

Also, intriguing results concerning the expression of proteasome immune subtypes were obtained. Although SDS PAGE and the following Western blotting showed approximately equal increase of LMP2 and LMP7 subunit content in the brain cortex of August rats compared to that of Wistar rats (Figure 2A and 2B), native PAGE detected different changes in the expression of various subtypes containing these subunits.

The most pronounced changes in the expression patterns belong to the immune subtypes containing LMP2 subunit. In the brain cortex of Wistar rats, the major quantity of LMP2 subunit is revealed in the proteasome structures bound to activator PA700, while in August rat cortex LMP2 subunit is mainly detected in proteasome structures bound to activator PA28ɑβ (Figure 5B and 5C). Since subunit LMP2 may be included in proteasome together with LMP7 or β5 subunit [16], it was important to compare the expression of all these subunits. The distribution of β5 subunit, but not LMP7 subunit, in gel zones I and II had similarities with that of LMP2 subunit (Figure 5B and 5C, Table 2). This indicates the decrease of the expression of subtypes β5-LMP2 bound to activator PA700 and increase of the expression of subtypes β5-LMP2 bound to activator PA28ɑβ in August rat cortex compared to that of Wistar rats. The content of activator PA28ɑβ was also increased in gel zone II corresponding to the sample of August rat cortex relatively that of Wistar rat cortex (Table 2). Detected changes were reflected in the proteasome pools, labeled by mAb to ɑ1,2,3,5,6,7 subunits, in gel zones I and II (Figure 5B and 5C, Table 2). In gel zone I, the quantity of subunits LMP7, β1 and Rpt6 is higher for the brain cortex of August rats (Figure 5B, Table 2) indicating the increase of the expression of proteasome subtype LMP7-β1 bound to PA700 activator. In gel zone II, the quantity of subunit LMP7 is the same for the brain cortex of August and Wistar rats (Figure 5B, Table 2). It shows the equal content of proteasome structures with subunit LMP7 and activator PA28ɑβ.

**Table 2:** Relative quantity of proteasome and activator subunits in brain cortex of August rats compared to that of Wistar rats

Proteasome subunit	Relative quantity, %	
	In gel zone I	In gel zone II
LMP2	59 ± 12*	213 ± 63*
LMP7	180 ± 32*	101 ± 10
β1	140 ± 41	60 ± 25*
β5	39 ± 16*	143 ± 20*
PA28ɑ	126 ±24	137 ± 16*
Rpt6	138 ± 14*	-
PA200	143 ± 23*	153 ± 27*
ɑ1,2,3,5,6,7	62 ± 10*	146 ± 23*

Data are presented as means and SEM. *Reliable difference from control magnitude at *p* < 0.05, n = 6.

The analysis of the expression patterns of constitutive proteolytic subunits β5 and β1 required our due attention. If we take into consideration the presence of these subunits only in immune subtypes β5-LMP2 and LMP7-β1, some results would be unclear. First, in gel zone I with the sample of August rat cortex the content of β5 subunit is decreased to a greater extent than the content of LMP2 subunit compared to that of Wistar rat cortex (Table 2). Second, in the same sample the increase of β1 subunit content is unreliable at *p* < 0.05 in contrast to LMP7 subunit content (Table 2). Third, in gel zone II with the sample of August rat cortex the content of β5 subunit is increased to a smaller extent than the content of LMP2 subunit compared to that of Wistar rat cortex (Table 2). Fourth, in the same zone the content of β1 subunit is reliably decreased in contrast to LMP7 subunit content (Table 2). Taken together, all these facts indicate the presence of the constitutive structures containing β5 and β1 subunits in both gel zones. The quantity of these structures is diminished in the brain cortex of August rats compared to that of Wistar rats.

The content of activator PA200 is enhanced in both gel zones corresponding to the brain cortex sample of August rats compared to that of Wistar rats (Figure 5B, Table 2). Note that proteasome structures containing activator PA200 or PA28ɑβ are revealed in the neighboring locations in the gel. This fact arises from the similar molecular mass of these activators, 200 kDa for activator PA200 and 196 kDa (28 kDa × 7) for activator PA28ɑβ.

It is interesting that the expression of the immune subunits in August rat cortex increases more significantly than the expression of the proteasome activators that was detected by both methods (Figure 2A and 2B, Table 2). Such blurred dynamics of the activator expression may be connected with two opposite processes in the cortex of August rats compared to Wistar rats: the increase of the expression of the immune proteasome subtypes bound to the activators and decrease of the expression of the constitutive proteasomes bound to these activators.

**Figure 6:**
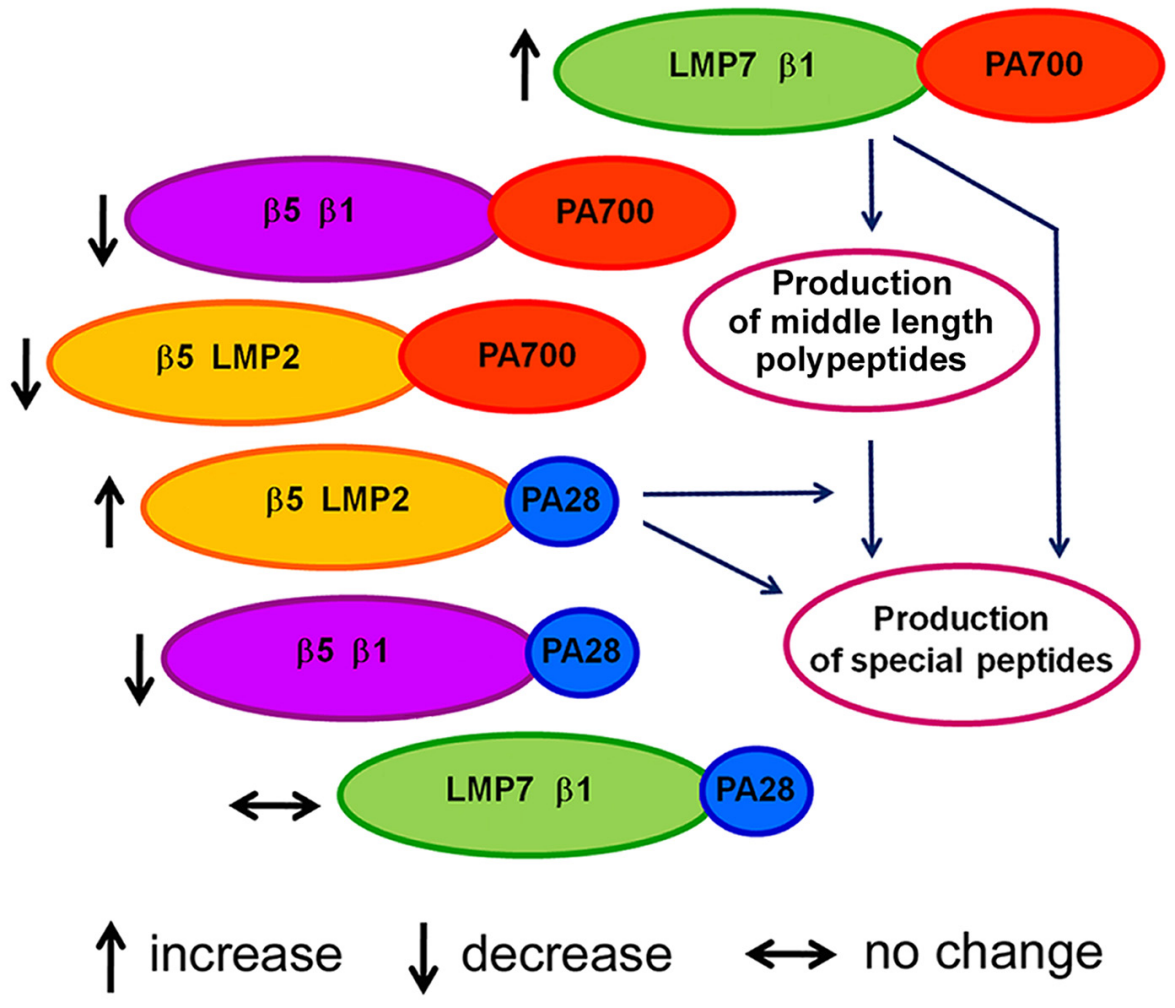
Scheme of changes in proteasome pool of August rat cortex compared to Wistar rat cortex

Thus, the data obtained demonstrate at least three proteasome subtypes differed in the combination of proteolytic subunits in the brain cortex of August and Wistar rats: LMP7-β1, β5-LMP2 and β5-β1. All these subtypes are bound to activator PA700 or activator PA28ɑβ. Detected changes in the expression of proteasome subtype groups in the cortex of August rats in comparison with that of Wistar rats are represented in Figure 6.

What do these changes mean? Based on the functions of the indicated subtypes, one may conclude that they provide the production of special biologically active peptides important for the development of adaptive processes under the stress evoked by monoamine metabolism deviations. Perhaps, the process of the peptide production is carried out in two steps. At first, proteasomes containing subunits LMP7, β1 and activator PA700 produce polypeptides of middle length from full size proteins in ubiquitin-dependent manner. Then the utilization of these polypeptides and formation of biologically active peptides from them are carried out by proteasomes containing subunits β5, LMP2 and activator PA28ɑβ. It is also possible that each of these immune proteasome subtypes is capable of individual production of special peptides. PA200 activator is likely to assist this process by the rapid release of hydrolysis products from the proteolytic cavity. Although the process of production of biologically active peptides in August rat cortex requires the separate investigation, it is already possible to assert the participation of detected immune proteasome subtypes in it.

### Expression patterns of proteasome subtypes in the brain stem and striatum of August rats

In spite of the changes of the proteasome pools in the striatum and stem of August rats in relation to Wistar rats are not as significant as in the cortex according to SDS PAGE and Western blotting, we analyzed the content of the immune subunits in these brain parts by native PAGE. It was shown that in August rat striatum, the content of LMP7 (but not LMP2) subunit was validly higher (by 80% at *p* < 0.05) in the proteasome structures bound to activator PA700, while the content of LMP2 (but not LMP7) subunit was validly increased (by 60% at *p* < 0.05) in the proteasome structures bound to activator PA28ɑβ (Figure 5E). In August rat stem, the reliably enhanced content (by 40% at *p* < 0.05) of LMP7 (but not LMP2) subunit in the proteasome structures bound to activator PA700 was detected (Figure 5G). This means that the enhanced expression concerns proteasome subtype containing proteolytic subunits LMP7 and β1 as well as subtype with proteolytic subunits β5 and LMP2 (but not subtype with subunits LMP7 and LMP2).

Although the quantities of the proteasome activators of the striatum and stem in August and Wistar rats do not reliably differ (Figure 2C–2F), it is possible that the increased content of proteasome immune subtypes bound to the activators is connected with the decreased content of the constitutive proteasomes bound to these activators.

Thus, despite the differences in the proteasome expression patterns in all studied brain areas of August rats, the increased expression is concerned with the same subtypes of the immune proteasomes, β5-LMP2-PA28ɑβ and / or LMP7-β1-PA700, indicating their important role in the maintaining of the neural plasticity.

Note that one more study of proteasome structure shows the existence of LMP2 subunit only in the combination with LMP7 subunit in proteasomes of some human organs [32]. Our results indicate the existence of LMP7 and LMP2 subunits in the different proteasome forms rather than in the common proteasome form in rat brain parts and they are in agreement with the study of M. Groettrup and coauthors [16].

### Proteasome activities in the brain parts of August and Wistar rats

**Figure 7:**
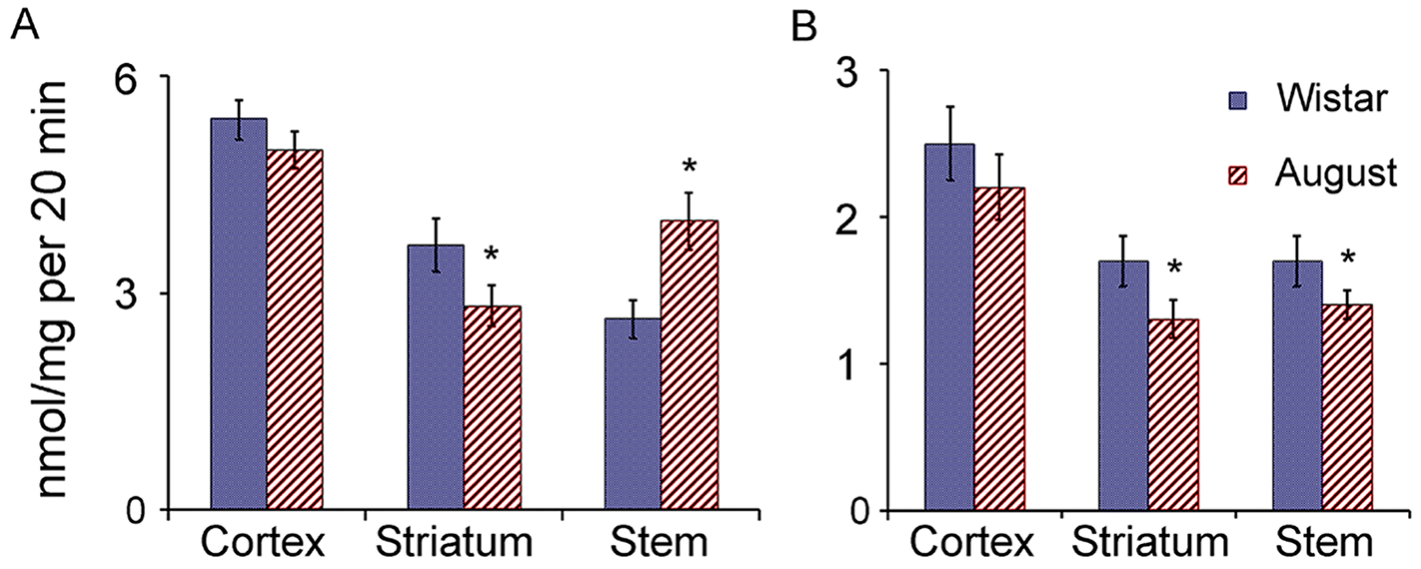
Proteasome ChTL and CL activities in brain parts of Wistar and August rats **(A)** ChTL activity, **(B)** CL activity. Error bars, SEM. *Reliable difference from corresponding activity of Wistar rats (*p* < 0.05, n = 8).

It is interesting that ChTL and CL activities in the brain cortex and striatum of August rats were slightly decreased or showed the tendency to decrease compared to that of Wistar rats (Figure 7). Only brain stem of August rats had the enhanced ChTL activity that correlated with the increased expression of LMP7 subunit displaying this activity. At the same time, in this brain area of August rats, CL activity was decreased by 20% in comparison with that of Wistar rats. On the whole, the changes in proteasome activities of the brain stem may be explained by the change of LMP7 subunit expression.

With regard to the cortex and striatum of August rats, the complex changes in the expression of the proteasome subunits and activators, compared to that of Wistar rats, did not significantly influence proteasome activities measured with the use of short substrates-oligopeptides. Most likely, the changes in proteasome pools in these brain areas of August rats are directed to the alteration of the specificity of hydrolysis of full-size and middle-size proteins with the maintenance of the ability to total cleavage of short substrates.

Although proteasome activities in the gel were detected for revealing the active proteasome zones, some additional interesting information was obtained. Proteasomes in gel zone I (26S proteasomes) possessed significantly higher ChTL activity in comparison with proteasomes bound to activator PA28ɑβ in gel zone II (Figure 5). It may be connected with the different content of LMP7 subunit which is known to display more pronounced ChTL activity compared to β5 subunit. CL activity was well revealed in both gel zones.

In gel zone I corresponding to the probes of all studied brain areas of August rats, ChTL activity was higher (by 25-30% at *p* < 0.05) and CL activity was slightly lower or showed the tendency to decrease compared to that of Wistar rats. In gel zone II corresponding to the probes of the cortex and striatum of August rats, CL activity was slightly enhanced in comparison with that of Wistar rats.

Thus, the data obtained indicate ChTL activity as the major activity for LMP7-β1-PA700 subtype group in all studied brain areas of August rats as well as CL activity as the major activity for β5-LMP2-PA28ɑβ subtype group in the cortex and striatum of August rats.

It is clear that proteasome activities, detected in a tube (Figure 7), reflected the mix of activities of multiple proteasome forms and did not reveal differences between them.

### Distribution and functions of the immune proteasomes in the brain

**Figure 8:**
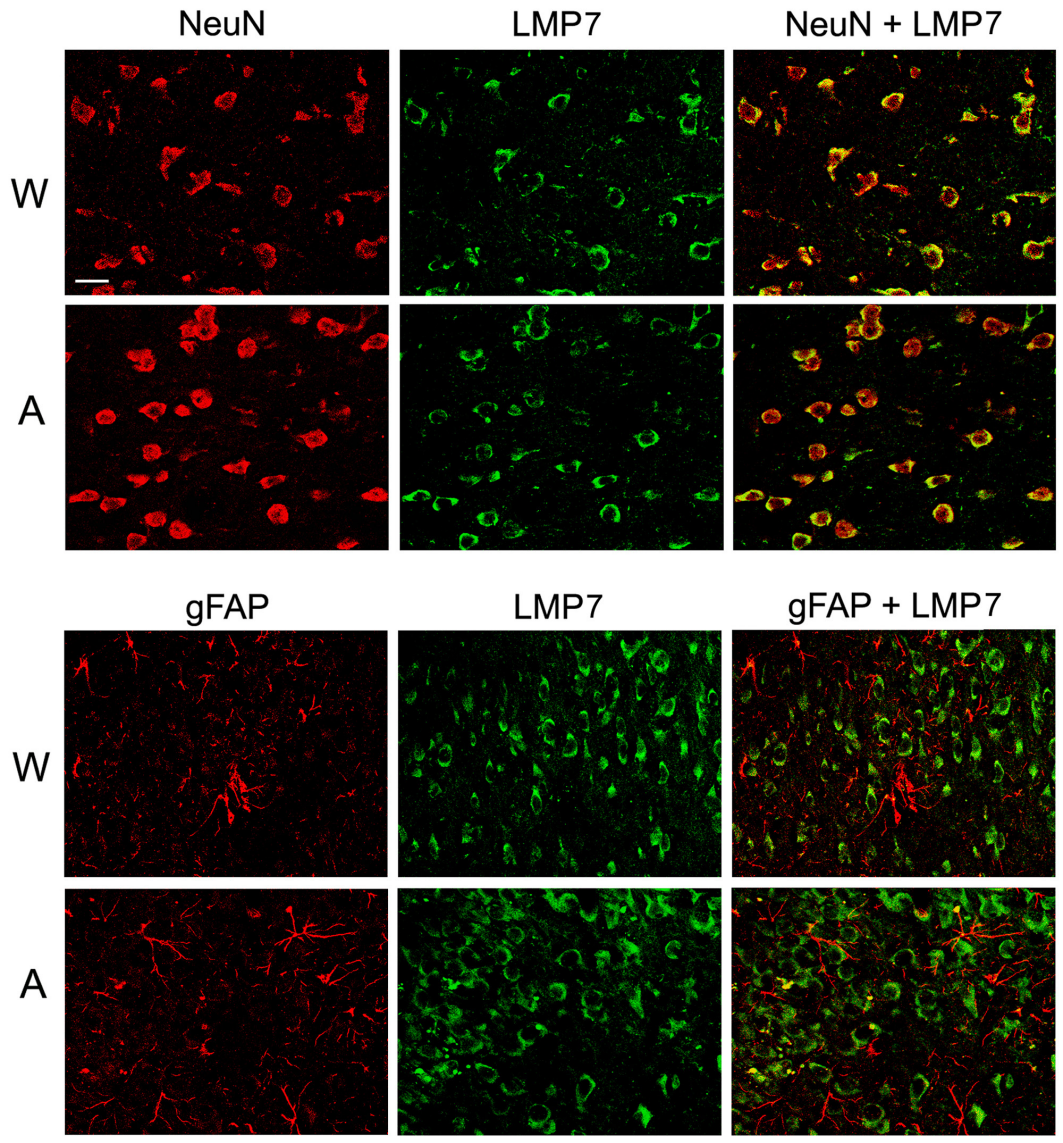
Distribution of proteasomes containing LMP7 subunit in brain cortex cells of Wistar and August rats Double immunofluorescent labeling of cells in brain cortex slices was performed with the use of mouse mAb to NeuN (marker of neurons) or gFAP (marker of glial cells) and rabbit pAb to LMP7 subunit. Scale bar, 10 μm. W, Wistar rats; A, August rats.

**Figure 9:**
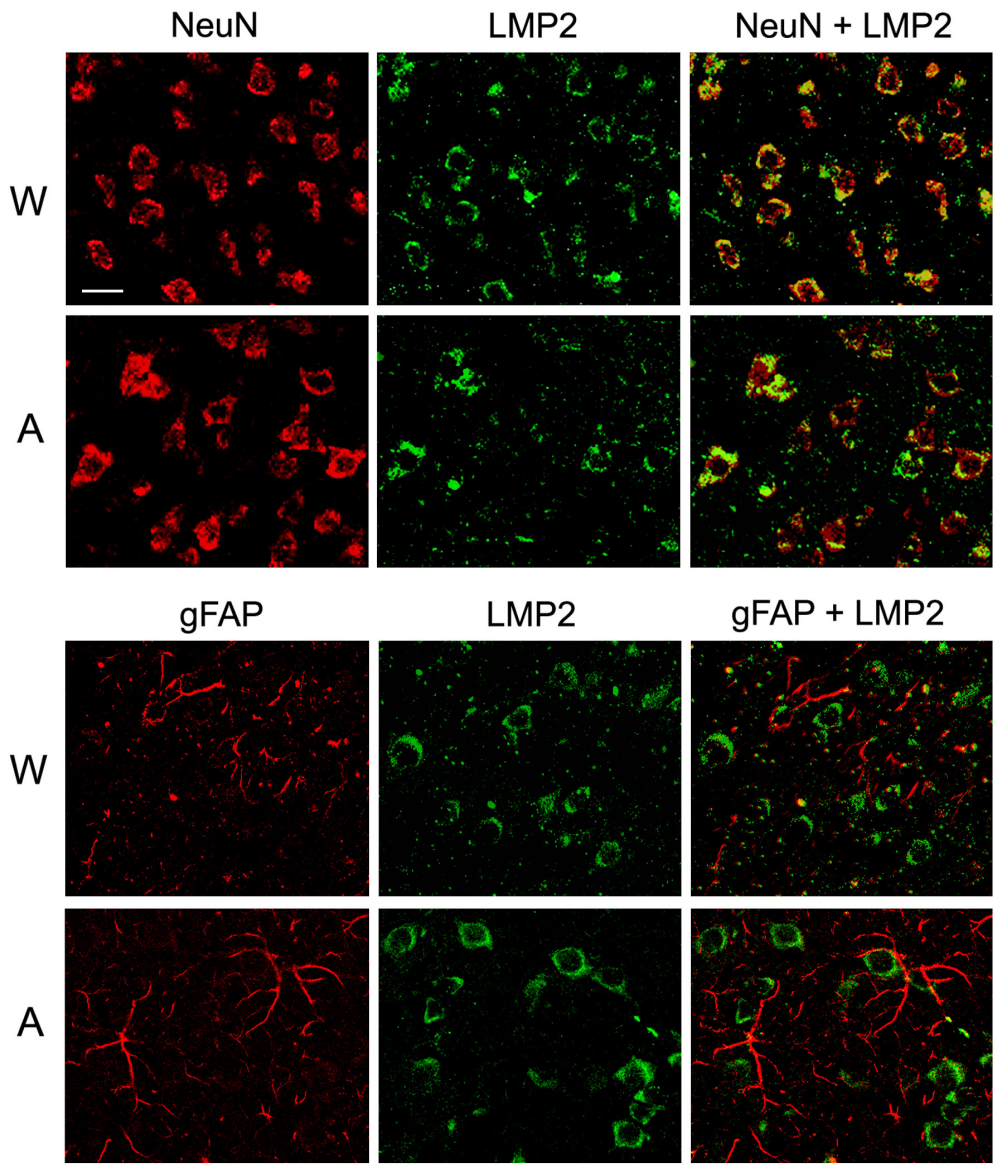
Distribution of proteasomes containing LMP2 subunit in brain cortex cells of Wistar and August rats Double immunofluorescent labeling of cells in brain cortex slices was performed with the use of mouse mAb to NeuN (marker of neurons) or gFAP (marker of glial cells) and rabbit pAb to LMP2 subunit. Scale bar, 10 μm. W, Wistar rats; A, August rats.

Double immunofluorescent labeling of brain cortex cells of Wistar and August rats by antibodies to cell markers and proteasome immune subunits LMP7 and LMP2 revealed both immune subunits in neurons, but not in glial cells (Figures 8 and 9) which may play the role of antigen-presenting cells under inflammation. So, this result indicates un-immune function of the immune proteasomes in the brain cortex of Wistar and August rats.

Together with MHC class I molecules, the immune proteasomes participate in the development of synaptic plasticity. The density of MHC class I molecules on pre- and post-synaptic membranes is of great importance because it regulates neuronal activity and, possibly, represents a structural basis for long term potentiation [33, 34]. MHC class I molecules serve as reporters of neurons transmitting the information to other neurons about products of proteolysis performed, predominantly, by the immune proteasomes. Besides, K. Ramachandran and S.Margolis discovered membrane proteasomes in mammalian nervous system that directly and rapidly modulated neuronal function by degrading intracellular proteins into extracellular peptides that could stimulate neuronal signaling [35].

It was earlier shown that the immune proteasomes were important for the regulation of the brain development [36] and adaptation of the brain to the stress provoked by the absence of MHC class I molecules [14]. In both these processes, the immune proteasomes is likely to generate the peptide or oligopeptide information for neuron-neuron interactions.

The pronounced immune functions of the immune proteasomes are displayed in the brain under neurodegenerative autoimmunity [37]. In oligodendrocytes of SJL mice with experimental autoimmune encephalomyelitis, the immune proteasomes containing LMP2 subunit are expressed in the enhanced quantity to generate significantly increased amounts of myelin basic protein peptides recognized by infiltrated cytotoxic lymphocytes. This leads to the loss of oligodendrocytes, axonal demyelination and development of neurodegeneration.

It would be interesting to discuss the deviations in proteasome systems of the brain areas, containing the enhanced content of dopamine and substantia nigra under Parkinson’s disease accompanied by the progressive loss of dopaminergic neurons in this brain area. For Parkinson’s disease, proteasome disfunction is known to be a major contributor to pathogenesis. There are decreased levels of core particle α subunits (the total proteasome pool) and PA700 activator, and dramatic diminishing of all three proteasome activities in the substantia nigra in sporadic Parkinson’s disease brains as compared with control brains [38–41]. For August rat brain areas with the enhanced content of dopamine, we discovered the increased or unchanged level of the total proteasome pool and its retuning on the special subtypes of the immune proteasomes. So, the opposite changes in dopaminergic system are related to the radically different changes in the proteasome pool.

Thus, the present study gives the additional information about un-immune functions of the immune proteasomes in the brain. Immune subtypes LMP2-PA28ɑβ and LMP7-PA700, detected in the enhanced quantities in August rat brain with the increased levels of monoamines, possibly produce special (oligo)peptides to provide the additional neuron-neuron interaction. Steady signaling between neurons may explain the high resistance of these rats to the acute emotional stress.

## MATERIALS AND METHODS

### Animals

The young males (two-month age) of inbred August rats with monoamine metabolism violations and Wistar rats (as a control) weighing 180-200 g were used. The rats were obtained from the Department of Animal Breeding of Research Institute of General Pathology and Pathophysiology of Russian Academy of Sciences. Experiments were carried out in accordance with the European Communities Council Directive of 24 November 1986 (86/609/EEC). All efforts were made to minimize suffering. All the protocols of manipulations with animals have been approved by the Commission on Bioethics of N.K. Koltsov Institute of Developmental Biology of Russian Academy of Sciences (Permit Number: 04.17/1).

Brain sections were obtained at 8-10°C according to Paxinos and Watson atlas [42]: frontal cortex (Bregma: from 5.0 mm up to 2.76 mm), striatum (Bregma: from 2.76 mm up to -3.0 mm), brainstem (Bregma: from -7.68 mm up to -14.64 mm). Brainstem area sections include parabracheal, oromotor, precerebelar, pontine reticular, facial raphe nucleus, etc.

### Antibodies

We used mouse monoclonal antibodies (mAb) to proteasome 20S **α**1,2,3,5,6,&7 subunits, Product Number: BML-PW8195; mouse mAb to proteasome 20S β5i (LMP7) subunit, Product Number: BML-PW8845; mouse mAb to proteasome 20S β1i (LMP2) subunit, Product Number: BML-PW8840; mouse mAb to proteasome 19S ATPase subunit Rpt6, Product Number: BML-PW9265; mouse mAb to proteasome 20S β1 subunit, Product Number: BML-PW8140; rabbit polyclonal antibodies (pAb) to proteasome 20S β5 subunit, Product Number: BML-PW8895; rabbit pAb to proteasome 20S β5i (LMP7) subunit, Product Number: BML-PW8356; rabbit pAb to proteasome 20S β1i (LMP2) subunit, Product Number: BML-PW8205; rabbit pAb to proteasome activator 11S α subunit, Product Number: BML-PW8185; rabbit pAb to HSC70/HSP70, (ADI-SPA-757) (all from Enzo Life Sciences, USA); rabbit pAb to PA200, sc-135512; mouse mAb to β-actin, sc-81178 (from Santa Cruz Biotechnology, USA); mouse mAb to gFAP, Catalogue Number: MAB360; mouse mAb to NeuN, Catalogue Number: MAB377 (EMD Millipore, USA); goat anti-rabbit IgG antibodies, labeled with Alexa 488, Catalogue Number: 11035; donkey anti-mouse IgG antibodies, labeled with Alexa 594, Catalogue Number: A11034 (Invitrogen, USA).

### High performance liquid chromatography with electrochemical detection

The dissected tissues of the brain parts were homogenized in 0.1 N HClO_4_ using an ultrasonic homogenizer (L-666, MSE, England). Then 10 μl of 0.2 N HClO_4_ containing 1 ng of 2,3-dihydroxybenzoic acid was added, followed by centrifugation at 15,200 g for 20 min. Supernatants were collected, frozen and stored at –70°C.

High performance liquid chromatography with electrochemical detection was used to measure the content of serotonin, dopamine and their metabolites in obtained supernatants. The samples were introduced into the injector (Raininn, USA) equipped with a 20 μl loop. The mobile phase consisted of 0.1 M citrate-phosphate buffer (pH 3.2) containing 0.3 mm sodium octanesulfonate, 0.1 mm ethylenediaminetetraacetic acid, and 8% acetonitrile (all chemicals, Sigma, USA). A flow rate of 800 μl/min was provided by a Gilson 10SC pump (France). Peaks of serotonin, 5-hydroxyindoleacetic acid, dopamine, 3,4-dihydroxyphenylacetic acid, and homovanillic acid were identified in accordance with elution time in the standard solution, and the substance amount was estimated as a ratio of the peak area of the internal standard solution to that of the specimen by using a software support (Multichrom 1.5, Ampersand Ltd.).

### Preparation of brain extracts

Extracts of the brain cortex, striatum and stem were prepared at 4°C. Tissues were mechanically homogenized in three volumes (w/v) of buffer containing 50 mM Na-HEPES, pH 7.5, 200 mM NaCl, and 10 mM EDTA. Homogenates were centrifuged under 18,000 g for 1 h. Obtained supernatants (extracts) were employed as samples for SDS PAGE, electrophoresis in native conditions and detection of proteasome activities.

### Electrophoresis in native conditions

All procedures were performed at 4°C. After the addition of sucrose (to 10%), extracts were subjected to the electrophoresis (10 μl, 40 μg of protein per lane) in a starting 3% polyacrylamide gel, prepared in 50 mM BisTris / Glycine buffer, pH 8.0 containing 1mM EDTA, and then in a 4–10% polyacrylamide gradient gel (acrylamide : bis-acrylamide, 19 : 1), prepared in the same buffer. Electrophoresis was performed at 60 V for 14 h, 140 V for 10 h and finally at 260 V for 20 h with utilizing the same buffer.

To visualize proteasome proteolytic activities, 200 mM Na-HEPES buffer, pH 7.5, containing 300 μM fluorogenic substrate N-succinyl-leu-leu-val-tyr-amido-4-methyl coumarin (Suc-LLVY-AMC) or Z-Leu-Leu-Glu-amido-4-methyl coumarin (Z-LLE-AMC) (1/20 of gradient gel volume) was placed to gel surface. The gel was covered by cellulose acetate film and incubated at 37°C for 20 min. Fluorescence bands in the gel were photographed in a dark room under illumination at 365 nm. Then the gel was incubated in the solution containing 0.025 M Tris, 0.19 M glycine, pH 8.3, and 10 M carbamide at the room temperature for 30 min under gentle agitation. This solution was also applied for the following semidry transfer of polypeptides onto Hybond-ECL membrane (Amersham). The use of carbamide instead of SDS for disruption of large protein complexes provides much better polypeptide transfer and allows us to obtain the reproducible results.

### Western blot analysis

After SDS electrophoresis in 13% polyacrylamide gel (15 μl of extract, 60 μg of protein per lane), polypeptides were transferred from the gel onto Hybond-ECL membrane (Amersham) by the standard procedure. After SDS and native PAGE, immunodetection was carried out with the use of primary antibodies to subunits a1,2,3,5,6,7, LMP7 (mAb), LMP2 (mAb) (1 : 1000), β1, β5, PA28a, Rpt6 (1 : 1500), and proteins PA200, HSP / HSC70s, (1 : 1500), β actin (1 : 1000) and corresponding secondary antibodies peroxidase conjugated. The image analysis was performed using standard “ImageJ” software. The relative quantities (optical density) of the immunoreactive bands on X-ray film were measured. The dependence of the optical density on the amount of the protein subjected to Western blotting was evaluated preliminarily. For further processing, portions were taken within a proportional dose range. Results were normalized to β actin content detected by Western blotting after SDS PAGE.

### Determination of proteasome activities

Proteasome ChTL activity and CL activity were determined by hydrolysis of fluorogenic substrates Suc-LLVY-AMC (Sigma, USA) and Z-LLE-AMC (Tebu-Bio, Belgium), respectively. The activity was determined in portions of 0.5–2 μl of brain extracts in final volume of 100 μl of reaction mix containing 50 mM Na-HEPES, (pH 7.5), 1 mM DTT, and 30 μM Suc-LLVY-AMC or Z-LLE-AMC.

In Supplementary Figure 1, the time dynamics of proteasome ChTL and CL activities in brain extracts of Wistar and August rats obtained with the use of DTX 880 Beckman Coulter and Multimode Analysis Software is presented. On the basis of the results showing the linear dependence of proteasome activities on reaction time, interval of 20 min was chosen for experiments.

So, the reactions were carried out at 37°C for 20 min and terminated by the addition of 1% SDS. The digestion product was detected by using a fluorimeter with the excitation wavelength of 380 nm and the emission wavelength of 440 nm. Proteasome-independent activity was determined in the presence of 5 μM of inhibitor of proteasome activities, Z-leucyl-leucyl-leucinal (MG-132) (Sigma, USA) (less than 10% activity in all samples) and subtracted from the values obtained in the absence of MG-132. In final, proteasome activities were normalized to 1 mg of protein, detected by Lowry method [43].

### Immunohistochemistry

Rats were anesthetized with pentobarbital (97.2 mg/kg i.p.), briefly perfused transcardially with saline and then with 4% paraformaldehyde in phosphate buffer (0.1 M, pH 7.4) for 5 min. Brains were dissected, post-fixed in buffered paraformaldehyde for 2 h at room temperature, equilibrated with 25% sucrose in phosphate buffer at 4°C, frozen, and cut into coronal 12-μm sections on slides using a cryostat Leica CM 1900. Brain structures of Wistar and August rats were mounted on the same slides.

For double staining, frontal cortex slices were blocked with 7% BSA and 0.3% Triton X100 in PBS for 1 h, and then subjected to antibody treatment. Antigen localization was determined by incubation of the slices with pAb to LMP2 or LMP7 (1 : 500) with 5% BSA and 0.1% Triton X100 in PBS overnight at 4°C. After incubation with the primary antibodies, slices were washed four times (5 min per wash) with PBS and treated with the goat secondary anti-rabbit IgG antibodies, Alexa 488-conjugated (1 : 800) for 2 h. After four washes with PBS (10 min per wash), the slides were incubated with other primary mAb to NeuN or gFAP (1 : 1000) with 5% BSA and 0.1% Triton X100 in PBS overnight at 4°C, washed four times (5 min per wash) with PBS and then treated with the donkey secondary anti-mouse IgG antibodies, Alexa 594-conjugated (1 : 700) for 2 h. After four washes with PBS (10 min per wash) slides were mounted with the Mowiol and analyzed under confocal microscope Leica SPE equipped with an Ar-Kr laser at the Core Facility on Cell Technologies and Optical Research Methods in Developmental Biology of N.K. Koltzov Institute of Developmental Biology of Russian Academy of Sciences. To ensure equal illumination for all treatments, the same intensity and filter settings were used throughout. Images were recorded at a resolution of 1024×1024 pixels and processed with the Leica LCS software. Control experiments were performed by omitting primary or secondary antibodies.

### Statistics

Results are presented as mean value of data measured for at least three parallel samples in one experiment and obtained in 6-8 experiments. Differences relative to control values were assessed using Kruskal-Wallis test of ANOVA and 95% confidence interval of the mean. Significant differences were considered to be at *p* < 0.05. The data were treated with F-test to compare variances. The statistical analysis program *Statistica 8.0* (Statsoft, 2008) was used.

## SUPPLEMENTARY MATERIALS FIGURE



## Abbreviations

HSP: heat shock protein
MHC: major histocompatibility complex
CL: caspase-like
ChTL: chymotrypsin-like
mAb: monoclonal antibodies
pAb: polyclonal antibodies
Suc-LLVY-AMC: N-succinyl-leu-leu-val-tyr-amido-4-methyl coumarin
Z-LLE-AMC: Z-Leu-Leu-Glu-amido-4-methyl coumarin
MG-132: Z-leucyl-leucyl-leucinal
