# Supplementary material for: Detection of active proteasome structures in brain extracts: proteasome features of August rat brain with violations in monoamine metabolism
Source: Oncotarget. 2017 Aug 10;8(41):70941–57. doi: 10.18632/oncotarget.20208 (PMC5642609; doi:10.18632/oncotarget.20208)
Supplement: Supplementary file 1 [file oncotarget-08-70941-s001.pdf]

Detection of active proteasome structures in brain extracts: proteasome features of August rat brain with violations in monoamine metabolism

SUPPLEMENTARY MATERIALS

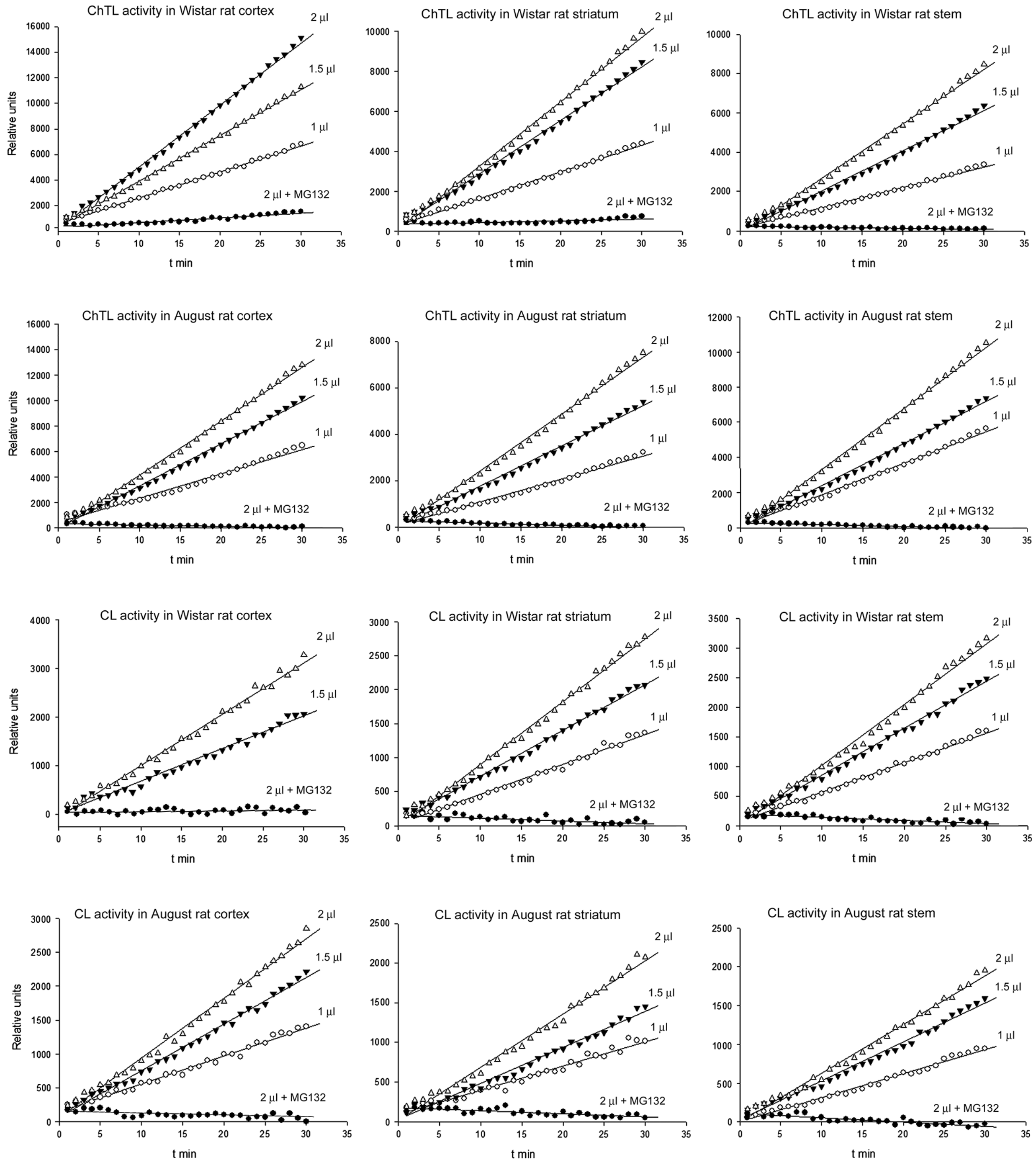

Supplementary Figure 1: Time dynamics of proteasome ChTL and CL activities in different volumes of brain extracts of Wistar and August rats.
